# The Effects of Inclisiran on the Subclinical Prothrombotic and Platelet Activation Markers in Patients at High Cardiovascular Risk

**DOI:** 10.3390/jcdd12090355

**Published:** 2025-09-16

**Authors:** Mateusz Maligłówka, Adrianna Dec, Łukasz Bułdak, Bogusław Okopień

**Affiliations:** Department of Internal Medicine and Clinical Pharmacology, School of Medicine in Katowice, Medical University of Silesia in Katowice, 40-007 Katowice, Poland

**Keywords:** inclisiran, PCSK9, heterozygous familial hypercholesterolemia, fibrinogen, coagulation factor VIII, PAI-1, PF-4, P-selectin

## Abstract

Atherosclerosis as a multifactorial disease remains the first cause of death worldwide. Current oral lipid-lowering drugs (especially statins) reduce low-density lipoprotein cholesterol (LDLC) levels in the blood, but their clinical efficacy seems to be partially attributed to pleiotropic effects on different pathophysiologic factors of atherosclerosis extending beyond lipid-lowering properties such as anti-inflammatory, antithrombotic and antioxidative features. Novel drugs that interfere with proprotein convertase subtilisin/kexin type 9 (PCSK9) axis of LDL-C receptors (LDLRs) degradation, from the group of monoclonal antibodies (e.g., alirocumab, evolocumab) or small interfering RNA (siRNA), e.g., inclisiran, are effective in reducing LDLC as well. However, data depicting their antithrombotic and antiplatelet activity are scarce, whereas prothrombotic properties of PCSK9 are widely described. Thus, we performed a study to assess the effects of inclisiran on subclinical prothrombotic [fibrinogen, coagulation factor VIII (FVIII), plasminogen activator inhibitor-1 (PAI-1)] and platelet activation markers (platelet factor-4 (PF-4), soluble *p*-selectin (sCD62P)). Ten patients at high cardiovascular risk with concomitant heterozygous familial hypercholesterolemia (HeFH)—study group 1, and fourteen patients at very high cardiovascular risk without concomitant HeFH—study group 2, were recruited for the study. Lipid profile, subclinical prothrombotic and platelet activation markers were assessed at the beginning and after 3 months of therapy with inclisiran. During therapy, statistically significant reductions in both study groups were seen in total cholesterol levels (study group 1: from 287.6 ± 94.2 to 215.2 ± 89.1 (mg/dL), *p* = 0.022; study group 2: from 211.7 ± 52.7 to 147.6 ± 55.4 (mg/dL), *p* < 0.001) and LDL-c (study group 1: from 180.8 ± 73.3 to 114.7 ± 71.5 (mg/dL), *p* = 0.031; study group 2: from 129.6 ± 46.8 to 63.4 ± 43.6 (mg/dL), *p* < 0.001). Lipid profile changes were associated with significant decrease in the concentration of FVIII in both groups (study group 1: from 33.3 ± 22 to 22 ± 14.5 (ng/mL), *p* = 0.006; study group 2: from 37 ±16.9 to 29.3 ±16.4 (ng/mL), *p* = 0.002) and fibrinogen, but only in study group 2 (from 51.4 (33.2–72.7) to 42.6 (31.3–57.2) (µg/mL), *p* = 0.035). Among platelet activation markers, a significant decrease in PF-4 in study group 2 was noted (from 286 (272–295.5) to 272 (268–281.5) (ng/mL), *p* = 0.047). However, there were no statistically significant changes in PAI-1 and sCD62P throughout the study. In our study, inclisiran appeared to be an effective lipid-lowering drug in patients at high cardiovascular risk. Moreover, it was shown that beyond lipid-lowering properties, the drug may also partially affect thrombogenesis and platelet activation.

## 1. Introduction

Atherosclerosis as a multifactorial disease resulting in accumulation of plaques in the subendothelial layer of middle- and large-sized arterial walls remains the principal cause of death worldwide [1,2,3]. In the pathogenesis of plaques formation, three main phases may be distinguished: initiation, progression and complications. Through all of them, different factors such as lipid accumulation, inflammation, endothelial dysfunction, platelet activation, thrombotic complications and oxidative stress play a significant role [2,4]. 

Atherosclerotic plaques as the segments of arterial walls with impaired function of endothelium are crucial in the activation of plasma coagulation cascade [5]. The exposure of tissue factor (TF) originated mainly from foam cells and smooth muscle cells (SMCs) to the plasma factor VII/VIIa, subsequently initiating thrombin and then fibrin production. Simultaneous platelet activation leads to the formation of platelet-rich thrombus [6]. Furthermore, endothelial cell injury stimulates the local, vascular expression of plasminogen activator inhibitor-1 (PAI-1) [7]. 

Proprotein convertase subtilisin/kexin type 9 (PCSK9), a member of the proprotein convertases family whose essential role in the pathogenesis of atherosclerosis had been initially attributed to the regulation of cholesterol metabolism, according to data from recent years, appeared to also be an important factor in thrombus and plaque formation as well as a regulator of platelet function [8,9]. 

At the beginning of atherosclerotic plaque formation, fatty streaks appear on the luminal surface of arteries. They are in fact cholesterol deposits, which tend to form earlier in patients with severe hypercholesterolemia. Heterozygous familial hypercholesterolemia (HeFH) is a relatively common genetic disease resulting in a premature formation of atherosclerotic plaques [10]. It is caused by different mutations in genes encoding proteins involved in lipid metabolism, e.g., receptors for low-density lipoprotein cholesterol (LDLRs), apolipoprotein B (apoB) or PCSK9. The disease manifests especially in early phases only with markedly elevated levels of low-density lipoprotein cholesterol (LDL-c) in plasma and can be diagnosed with the Dutch Lipid Clinic Network (DLCN) criteria consisting of LDL-c, history of cardiovascular (CV) events (including family history), symptoms of disease in physical examination and genetic analysis [11]. 

Nevertheless, some data suggest that high cardiovascular risk in patients with HeFH may partially stem from higher levels of plasma coagulation factors (e.g., fibrinogen, factor VIII (FVIII)) as well as increased platelet activation markers (e.g., mean platelet volume (MPV), platelet factor 4 (PF-4), CD40L) [12,13,14,15]. Such prothrombotic features, also typical for patients with a history of cardiovascular events (e.g., myocardial infarction, stroke) without concomitant HeFH, are considered to represent residual cardiovascular risk factors [16]. High level of PCSK9 in patients with HeFH (e.g., gain-of-function mutations of PCSK9) as well as in patients at very high cardiovascular risk (with a history of cardiovascular events), which may be additionally raised by statin therapy, seems to be partially responsible for increasing plasma thrombogenicity and platelet activation. Moreover, loss-of-function mutations of PCSK9 and enzyme inhibition by monoclonal antibodies appeared to lower cardiovascular risk [17].

Current oral lipid-lowering drugs (e.g., statins and ezetimibe) effectively reduce low-density lipoprotein cholesterol (LDLC) levels [18,19]. Furthermore, they have also been able to decrease the mortality rate and number of acute cardiovascular events [19,20]. Such beneficial influence on cardiovascular risk may be partially attributed to their pleiotropic properties beyond lipid-lowering effects such as reducing inflammation and oxidative stress, improving platelet and endothelial function or antithrombotic features [21,22].

A relatively new group of hypolipemic drugs that interfere with PCSK9 axis for LDLC receptors (LDLRs) degradation appeared to be a very efficient tool in the therapy of patients at high cardiovascular risk [23].

Monoclonal antibodies against circulating form of PCSK9 (e.g., alirocumab, evolocumab) and small-interfering ribonucleic acid (siRNA)—inclisiran that inhibits the production of PCSK9 in the liver cells—were implemented in the guidelines by European Society of Cardiology (ESC) with strong recommendation as an additional lipid-lowering therapy in patients with atherosclerotic complications (e.g., ischemic heart disease, peripheral artery disease, ischemic stroke) who did not meet LDL-c target [24,25,26,27]. The detailed review of inclisiran’s pharmacological features was published by Dec A. et al. in 2023 [28].

Taking into account pleiotropic effects of oral lipid-lowering drugs (mainly statins), pathogenetic factors of atherosclerosis such as thrombosis and platelet activation that are possibly linked with the function of PCSK9 in patients at high and very high cardiovascular risk, as well as the still lacking data concerning extralipid effects of inclisiran (the silencer of PCSK9 formation), we conducted a study which assesses its possible impact on subclinical prothrombotic (e.g., fibrinogen, FVIII, PAI-1) and platelet activation markers (e.g., PF-4, sCD62P) of atherosclerosis after 3 months of therapy. Furthermore, the study was carried out to determine the presumptive differences in the response to the therapy depending on the etiology of atherosclerosis (HeFH vs. non-HeFH) and to define possible pleiotropic effects of the drug. 

## 2. Materials and Methods

### 2.1. Study Population

The study was performed between June 2023 and November 2024 in the Outpatient Department for the Treatment of Metabolic Diseases belonging to the Internal Medicine and Clinical Pharmacology Ward of the University Clinical Center of Prof. K. Gibiński of the Medical University of Silesia in Katowice. There were 24 patients (13 women and 11 men) at ages between 41 and 81 years old—who did not achieve LDLC plasma target level (according to 2021 ESC Guidelines on cardiovascular disease prevention in clinical practice), met inclusion criteria and did not meet exclusion criteria—who participated in this study. 

The inclusion criteria consisted of the following: age over 18 years old; concomitant hypercholesterolemia with high cardiovascular risk (HeFH without a history of cardiovascular events, i.e., acute coronary syndrome, coronary or other arterial revascularization, stroke) or very high cardiovascular risk (HeFH with a history of cardiovascular events; polygenic hypercholesterolemia with a history of cardiovascular events); exceeded therapeutic goal (LDL-c over the required level characteristic to the specific cardiovascular risk groups according to ESC guidelines); prior treatment with statin at high doses for at least 3 months (atorvastatin 40–80 mg per day or rosuvastatin 20–40 mg per day); in case of statin intolerance at high dose, prior statin treatment at highest tolerated dose for at least 3 months; prior ezetimibe treatment for at least 1 month; alanine aminotransferase (ALT) or aspartate aminotransferase (AST) < 3 upper limit level; creatine kinase (CK) < 5 upper limit level; thyroid-stimulating hormone (TSH) level at normal range; and obtained written informed consent. The exclusion criteria consisted of the following: age under 18 years old; secondarily originated hypercholesterolemia; diabetes mellitus (DM); liver failure (class B–C according to Child–Pugh classification), decompensated heart failure (grade III–IV according to the New York Heart Association); history of oncological treatment within 5 years; inflammatory diseases; current anticoagulation treatment; poor compliance; lack of informed consent; pregnancy or breastfeeding; history of hypersensitivity reactions to PCSK9 monoclonal antibodies or inclisiran; and disturbances in laboratory results as follows: ALT or AST > 3 upper limit level or bilirubin > 1.2 mg/dL, triglycerides (TG) > 500 mg/dL, hemoglobin < 10 g/dL or >17 g/dL, red blood cell count < 3.5 M/μL or >5.5 M/μL, white blood cell count < 3.5 K/μL or >10 K/μL, and platelet count < 140 K/μL or >400 K/μL.

**Figure 1:**
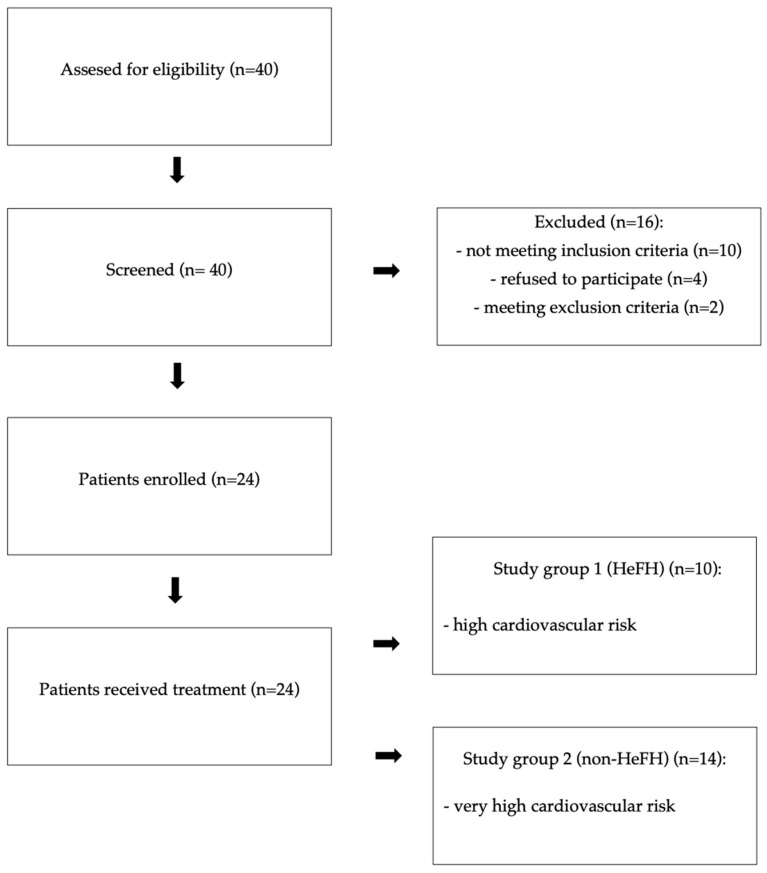
Study flow chart.

In study group 1, there were 10 patients (8 women, 2 men) diagnosed with HeFH (scoring at least 9 points according to DLCN) at high cardiovascular risk (HeFH without a history of cardiovascular events). In study group 2, there were 14 patients (5 women, 9 men) at very high cardiovascular risk with polygenic hypercholesterolemia (scoring less than 9 points according to DLCN) and a history of cardiovascular events. Figure 1 presents the study flow chart. 

### 2.2. Ethics

Written informed consent in accordance with the Helsinki Declaration was taken in advance from each patient. The study protocol was accepted by Medical University of Silesia’s Bioethical Committee (BNW/NWN/0052/KB1/53/23) (13 June 2023).

### 2.3. Assessment of Laboratory Parameters

Laboratory blood tests included complete blood count, MPV, creatinine, AST, ALT, gamma-glutamyl transferase (GGT), alkaline phosphatase (ALP), bilirubin, international normalized ratio (INR), activated partial thromboplastin time (APTT), glucose, glycated hemoglobin (HbA1c), creatine kinase (CK), total cholesterol (TC), LDL-c, HDL-c, TG, TSH, N-terminal prohormone B-type natriuretic peptide (NT-proBNP), and subclinical prothrombotic and platelet activation markers, namely fibrinogen, FVIII, PAI-1, sCD62P and PF-4. The plasma measurements were performed just before the first injection of inclisiran and after three months, just before the second dose of the drug. 

Basic laboratory analyses were made with the use of commercially available equipment (RAPIDPoint 500, Siemens Healthineers, Erlangen, Germany; Sysmex XN1000, Sysmex Corp., Kobe, Japan; Cobas PRO, Roche Diagnostics, Basel, Switzerland; ACL 500, Instrumentation Laboratory, Bedford, MA, USA). The analyses of subclinical prothrombotic and platelet activation markers were performed with the use of respective ELISA detection kits (ELISA Kit for Fibrinogen (FG) Cloud-Clone Corp., Houston, TX, USA; ELISA Kit for Coagulation Factor VIII (FVIII) Cloud-Clone Corp., Houston, TX, USA; ELISA Kit for Plasminogen Activator Inhibitor 1 (PAI-1) Cloud-Clone Corp., Houston, TX, USA; Human CD62P ELISA Kit Diaclone SAS, Besancon, France; ELISA Kit for Platelet Factor 4 (PF4), Cloud-Clone Corp., Houston, TX, USA) and xMarc Microplate Absorbance Spectrophotometer (Bio-Rad Laboratories, Inc. Hercules, CA, USA).

### 2.4. Statistical Analyses

The necessary sample size was estimated using previous experimental data on the impact of rosuvastatin on FVIII activity, which reduced the FVIII activity by around 11.1% during four-week therapy in a group of patients with cardiovascular risk factors [29]. Assuming 7.5% standard deviation of differences, a sample size of *n* = 8 per group would be required to achieve 80% power with a two-sided alpha level of 0.05. All collected data were collated in a Microsoft Excel 365 spreadsheet (Microsoft Corp., Redmond, WA, USA) and processed in the Statistica software package v. 13.0 (StatSoft Inc., Tulsa, OK, USA) and Plus Set v. 5.0 (TIBCO Software Inc., Palo Alto, CA, USA). The Shapiro–Wilk’s test was used to assess the normality of distribution. Data with normal distribution are presented in tables as mean with standard deviation (SD). Data with non-normal distribution are shown as median with interquartile range (IQR). The differences concerning baseline parameters and those describing response to the therapy with inclisiran between both study groups were measured with Welch’s *t*-test (with means for normally distributed data) and Mann–Whitney test (with medians for non-normally distributed data). The statistical differences concerning continuous data before and after treatment with inclisiran were assessed with Student’s *t*-test (with means for normal distribution) and the Wilcoxon (with medians for non-normal distribution). A chi-square with Yates’s correction test was performed to examine the differences in categorical variables. A statistical significance was indicated with a value of *p* < 0.05.

## 3. Results

A total of 24 patients were included in the study and assigned to two study groups. In study group 1, there were 10 patients (8 women, 2 men), at high cardiovascular risk with concomitant HeFH, whereas in study group 2, there were 14 patients (5 women, 9 men) at very high cardiovascular risk with no HeFH. There was no statistically significant difference between study groups concerning gender distribution (*p* = 0.083). Patients from study group 1 were statistically younger than patients from study group 2: 54 years old (±10 y. o.) vs. 63 years old (±10 y. o.) (*p* = 0.032). Furthermore, 30% of participants from study group 1 and 93% from study group 2 were being treated with antiplatelet drug (acetyl salicylic acid). The difference was statistically significant at *p* = 0.005. The detailed baseline characteristics of patients are presented in Table 1.

Basic laboratory parameters assessed at the initiation of the study showed significant differences in total cholesterol (TC) concentration (study group 1: 287.6 ± 94.1 vs. study group 2: 211.7 ± 52.7 (mg/dL) (*p* = 0.038)), whilst differences in LDL-c that appeared to be relatively high (study group 1: 180.8 ± 73.3 vs. study group 2: 129.6 ± 46.8 (mg/dL)) did not reach statistical significance (*p* = 0.072). Furthermore, in patients from study group 1, fasting glucose levels were significantly lower in comparison with study group 2 (93.9 (89–95.3) vs. 102 (98.5–106) (mg/dL) (*p* = 0.009)), which was subsequently followed by nearly statistically significant differences in HbA1c levels between two study groups (study group 1: 5.6 (5.2–5.7) vs. study group 2: 6 (5.6–6.1) (%) (*p* = 0.053)).

Initially, there were no statistically significant differences between both study groups in the levels of subclinical prothrombotic and platelet activation markers or values of INR and APTT mean platelet volume (MPV). Table 2 presents baseline laboratory findings in study groups. 

**Figure 2:**
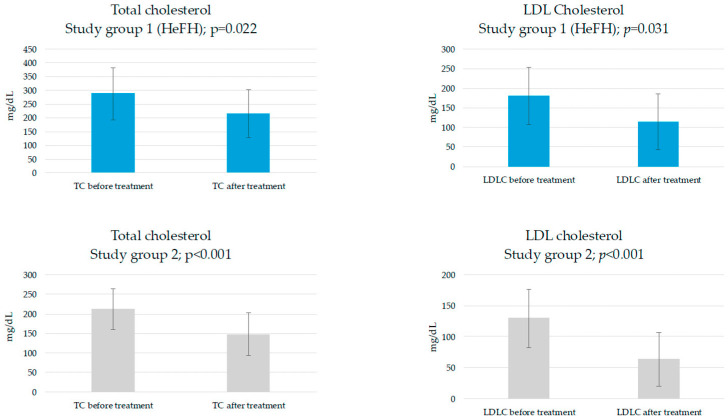
Effects of inclisiran on TC and LDL-c levels.

After 3 months of therapy, statistically significant reductions in TC level were observed (study group 1: from 287.6 ± 94.2 to 215.2 ± 89.1 (mg/dL), *p* = 0.022; study group 2: from 211.7 ± 52.7 to 147.6 ± 55.4 (mg/dL), *p* < 0.001) and LDL-c (study group 1: from 180.8 ± 73.3 to 114.7 ± 71.5 (mg/dL), *p* = 0.031; study group 2: from 129.6 ± 46.8 to 63.4 ± 43.6 (mg/dL), *p* < 0.001) (Figure 2). Nonetheless, the magnitude of reduction did not achieve statistical significance in either lipid parameters (study group 1: ΔTC = −72.4 ± 82.8 (mg/dL), study group 2: ΔTC = −64.1 ± 33.8 (mg/dL), *p* = 0.769; study group 1: ΔLDL-c = −66.1 ± 81.9 (mg/dL), study group 2: ΔLDL-c = −66.2 ± 27.1 (mg/dL), *p* = 0.997)). The target of LDL-c below 70 mg/dL for the patients with high cardiovascular risk in accordance with ESC guidelines was achieved in study group 1 by 10% of patients and in study group 2 by 42.9% of patients. Nevertheless, such difference was not statistically significant at *p* = 0.242. Furthermore, there were no significant changes concerning values of INR, APTT and MPV in either study groups.

Analysis of subclinical prothrombotic markers evaluated in the study showed significant decrease in the concentration of FVIII in both groups (study group 1: from 33.3 ± 22 to 22 ± 14.5 (ng/mL), *p* = 0.006; study group 2: from 37 ± 16.9 to 29.3 ± 16.4 (ng/mL), *p* = 0.002). However, there were no differences between groups in the extent of reduction (study group 1: ΔFVIII = −5.4 (−18.6–−2.8) (ng/mL); study group 2 ΔFVIII = −8.1 (−9.8–−0.9) (ng/mL), *p* = 0.446). Furthermore, a significant drop was observed in the concentration of fibrinogen but only in study group 2 (from 51.4 (33.2–72.7) to 42.6 (31.3–57.2) (µg/mL), *p* = 0.035). There were no differences in the concentration of PAI-1 in both study groups. 

**Figure 3:**
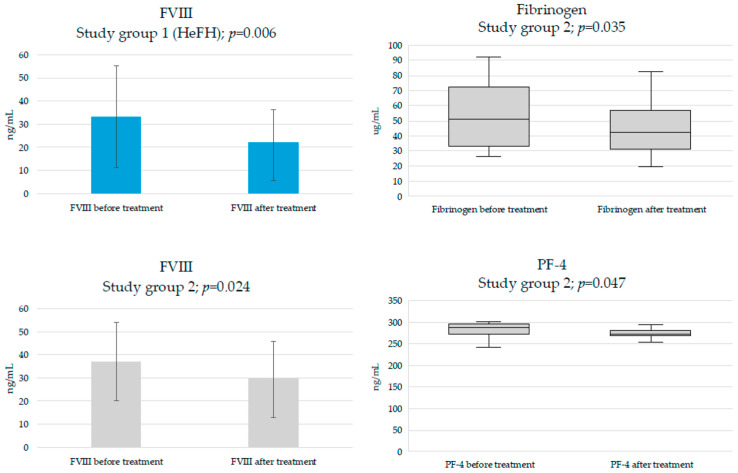
Effects of inclisiran on prothrombotic and platelet activation markers.

In the group of platelet activation markers, a significant decrease in PF-4 in study group 2 (from 286 (272–295.5) to 272 (268–281.5) (ng/mL), *p* = 0.047) was noted. The concentration of P-selectin was not statistically different in either of the groups. Figure 3 presents significant changes in the concentration of prothrombotic and platelet activation markers after the therapy with inclisiran.

There were no statistically significant changes in the levels of bilirubin, ALT, AST, creatinine, NT-proBNP, TSH, WBC, RBC, HGB nor creatine kinase in both study groups. No minor or major adverse effects were recorded during the course of the study.

## 4. Discussion

In our previously published article, we focused on the effects of inclisiran on subclinical markers of inflammation in atherosclerosis among patients at high cardiovascular risk [30]. In this paper, we have investigated a potential influence of the drug on different pathophysiologic factors of atherosclerosis beyond inflammation and lipids that are considered to be responsible for residual cardiovascular risk, namely thrombosis and platelet activation.

The differences between two study groups at the beginning of the study included age, concentration of TC and fasting glucose level. Although patients from study group 1 were younger than those from study group 2, due to diagnosed HeFH, they represent high cardiovascular risk according to ESC guidelines [27]. Mutations in genes responsible for lipid metabolism might reflect significant differences in TC levels between study groups and almost significant differences in LDL concentration [10]. Furthermore, carbohydrate metabolism impairment that is associated with aging, may mirror higher glucose levels in older patients from study group 2 [31].

The significant difference between study groups concerning the current treatment with antiplatelet agent results from more frequent incidence of cardiovascular events in medical history of patients from study group 2 and the necessity for introducing secondary prevention treatment [32].

Lipid changes after three months of therapy with inclisiran, manifested by TC and LDLC reductions in both groups, were similar to those observed in randomized controlled trials (RCTs), e.g., ORION-3, ORION-9 [33,34]. What is noteworthy is that one dose of the drug enabled LDL levels below 70 mg/dL in 8 out of 24 patients. 

PCSK9 is one of the family members of serine proteases discovered by Seidah et al. in 2003, and it is responsible mainly for the degradation of LDLRs on the surface of hepatic cells [35]. The soluble protease after binding to the epidermal growth factor homology domain A (EGF-A) of LDLRs precludes their attachment to the LDLC particles. Furthermore, PCSK9 promotes the degradation of LDLRs and inhibits their recycling also in the intracellular pathway [36]. This results in disturbances in cholesterol transportation between blood and liver and subsequent increase in LDLC plasma concentration [37].

Outside the basic impact on liver LDLC uptake, PCSK9 is associated with the initiation, progression and complications of atherosclerosis such as vascular inflammation, platelet functions or thrombogenesis [38]. PCSK9 stimulates proinflammatory cytokines secretion from macrophages and increases ROS production in mitochondria that may be harmful for endothelial cells [39,40].

Prothrombotic effects of PCSK9 and its association with platelet activation are widely described both in *humans* and *rodents*. Furthermore, the mechanisms in which above-mentioned serine protease affects hemostasis, are only partially connected with systemic lipid changes. It may also directly regulate platelet signaling and coagulation cascade [41].

Studies in *mice* revealed lower risk for venous thrombosis in individuals with PCSK9 deficiency in comparison with wild-type ones (the presence of inferior vena cava thrombosis: 25% vs. 60%, *p* < 0.05) [42]. In humans, circulating PCSK9 levels were positively correlated with levels of fibrinogen (*p* < 0.037) and negatively correlated with prothrombin time (*p* < 0.001) [43,44]. Moreover, data suggest possible positive correlation between PCSK9 and fibrinolytic processes characterized by the level of plasminogen activator inhibitor-1 (PAI-1) [45].

Positive association between PCSK9 levels and platelet reactivity was described in studies on humans and mice [46,47]. Direct activation takes place mainly by binding the CD36 platelets’ antigen with activation of signaling pathway and subsequent release of secondary transmitters and markers of platelet activation, e.g., soluble P-selectin (sCD62P), PF-4 or soluble CD40L [38,48,49,50,51].

The assessment of inclisiran’s influence on prothrombotic markers after three months of treatment revealed statistically significant reduction in FVIII concentration in both groups. High levels of FVIII is considered as a risk factor for venous and arterial thrombosis [52]. Studies in *mice* showed that up-regulation in the number of LDLRs results in accelerated clearance of FVIII from circulation [53]. Moreover, lipid-lowering therapy based on statins appeared to be effective in reducing FVIII concentration [29,54].

Studies in *mice* confirmed the ability of alirocumab to lower the levels of FVIII [55]. Furthermore, the post hoc analysis of RCTs (FOURIER, ODYSSEY) in *humans* treated with evolocumab and alirocumab revealed their ability to reduce the risk for venous thromboembolism (*p* = 0.007) [56]. Nevertheless, data concerning the direct effects of PCSK9 inhibitors (PCSK9is), including inclisiran on FVIII levels in *humans*, are scarce. To the best of our knowledge, this study, as a first one, presents the drop of FVIII concentration after therapy with inclisiran. However, further research is necessary to unequivocally establish the possible influence of inclisiran on FVIII levels and subsequent clinical implications. 

Fibrinogen plays a crucial and complex role in pathophysiology of atherosclerosis as an acute phase protein (marker of inflammation), a coagulation factor I and a cofactor in platelet activation [57]. Moreover, levels of PCSK9 are positively correlated with fibrinogen concentration [43]. Data concerning the effects of antibodies against PCSK9 (PCSK9 mabs) on fibrinogen levels are limited to small-group studies and is divergent [58,59]. The effect of inclisiran on fibrinogen level in our study also seems ambiguous because the significant drop was only seen in patients from study group 2. Data from large-group research are needed to conclusively explore the possible interactions between inclisiran and fibrinogen.

PAI-1, by decreasing the plasma fibrinolytic activity which plays a protective role against thrombosis in arterial vessels, appears to be involved in pathophysiology of atherosclerosis and increases the risk for ischemic cardiovascular events [60]. To date, one study showed reducing effect of alirocumab on the concentration of PAI-1 [59]. Nonetheless, our research revealed no significant changes in PAI-1 levels after 3 months of therapy with inclisiran. 

The role of platelets in acute atherosclerotic events, e.g., myocardial infarction or stroke resulting from plaque ruptures, is well established [61]. However, increasing evidence based on animal and human studies also confirms their participation in earlier stages of plaques formation and growth [62].

Platelets activated by high LDL-c predispose the formation of platelet–leukocyte complexes mediated by the presence of surface proteins, e.g., CD62P or GPIIb/IIIa via fibrinogen [63,64,65]. Their subsequent adhesion to endothelial cells and translocation through arterial wall results in the production of proinflammatory chemokines (e.g., platelet factor-4 [PF-4]), interleukins (e.g., IL-18) and CD40L that promote monocyte recruitment, therefore intensifying the progression of atherosclerotic plaques growth [64,65,66,67,68,69,70].

Platelet activation plays an indisputable role in the pathogenesis of atherosclerosis, and its transmitters seem to be a future therapeutic target for patients with high cardiovascular risk [71]. Several mediators of platelet activation are involved in the initiation of plaque growth (e.g., CD62P) and can intensify the inflammation and thus progression of atherosclerosis (e.g., PF-4) [65,69]. Furthermore, PCSK9 appeared to be positively associated with levels of secondary platelet activation transmitters, e.g., by activation of their release [38]. Analysis of RCTs with alirocumab and evolocumab confirmed their positive effect in decreasing the levels of sCD62P (*p* = 0.043) and PF-4 (*p* = 0.046) [72]. In our study, therapy with inclisiran significantly reduced the concentration of PF-4 but only in study group 2. Levels of sP-selectin did not change. 

The selective effects of inclisiran on subclinical thrombosis and platelet activation markers observed in our study require further research to be confirmed and to explicitly explain the mechanisms in which the drug may influence the residual risk factors of atherosclerosis. The greater impact on plasma and platelet markers of subclinical thrombosis in patients from study group 2 consisting of older participants, with a history of cardiovascular complications, might be attributed to a more advanced stage of atherosclerotic plaque development and higher platelet reactivity resulted from above-mentioned patients’ features [61,73]. On the other hand, participants on current acetylsalicylic acid treatment from study group 2 were more frequent. Nevertheless, above data suggest that lipid-lowering therapy with inclisiran may be effective in the context of LDL-c reduction. Its partial influence on coagulation and platelet function may suggest lower potential for evoking bleeding complications in patients at high cardiovascular risk. 

Taking into consideration lack of alterations observed in the function of major organs (e.g., liver, kidneys, heart, bone marrow, thyroid gland) after one dose of inclisiran in basic laboratory parameters presented in Table 2 and data from RCTs, the drug seems to be safe [33,74]. Nonetheless, longer observation is necessary to unequivocally confirm this observation. 

The limitations that have to be taken into consideration when analyzing the results of our study involve the small number of patients included in the research in a short space of time. The study involved only Caucasians, and the difference in age and glucose levels between study groups could interfere with the results. Data concerning concomitant diseases, addictions or demographic features that might affect the findings were not accessible. Due to financial limitations, the levels of lipoprotein(a) were not checked during the study. Furthermore, it was a single-center study without a control group due to ethical issues. All participants with high cardiovascular risk and indications for the escalation of lipid-lowering therapy were treated with inclisiran. 

## 5. Conclusions

In our study, inclisiran appeared to be an effective tool as an additional lipid-lowering drug in patients at high and very high cardiovascular risk. Moreover, it was shown that beyond lipid-lowering properties, the drug may also partially affect thrombogenesis and platelet activation—factors considered to be responsible for residual cardiovascular risk, markedly expressed in patients with HeFH and a history of cardiovascular events, and causatively linked with the level of PCSK9. Further research including RCTs is needed to explicitly explore the pleiotropic effects of inclisiran on the course of atherosclerosis and possible clinical benefits especially in the context of cardiovascular risk reduction. 

## Figures and Tables

**Table 1:** Baseline characteristics of patients.

	Study Group 1 (HeFH); *n* = 10	Study Group 2; *n* = 14	Statistical Test	*p* Value
**Age, years**	54 ± 10	63 ± 10	Welch’s *t*-test	**0.032**
Women, %	80	35.7	Chi-square with Yates’s correction test	0.083
**Current antiplatelet** **therapy, %**	30	93		**0.005**

**Table 2:** Baseline laboratory findings in study groups.

	Study Group 1 (HeFH); *n* = 10	Study Group 2; *n* = 14	Statistical Test	*p* Value
WBC, K/μL	5.9 ± 1.4	6.4 ± 1.1	Welch’s *t*-test	0.333
RBC, M/μL	4.6 ± 0.4	4.8 ± 0.6		0.333
HGB, g/dL	13.7 ± 1.5	14.7 ± 1.4		0.126
PLT, K/μL	247.6 ± 68.9	247.9 ± 42.4		0.99
MPV, fL	10.7 ± 1	10.4 ± 1.2		0.534
**TC, mg/dL**	287.6 ± 94.1	211.7 ± 52.7		**0.038**
LDLC, mg/dL	180.8 ± 73.3	129.6 ± 46.8		0.072
HDLC, mg/dL	57.5 (51.2–121.8)	47.6 (41–59.6)	M-W	0.064
TG, mg/dL	117.3 ± 50.8	144.9 ± 50.8	Welch’s *t*-test	0.206
Total bilirubin, mg/dL	0.5 ± 0.2	0.7 ± 0.3		0.043
ALT, U/L	26 ± 13.8	33 ± 16.9		0.28
AST, U/L	24.7 (23.1–26.7)	26 (19–31.1)	M-W	0.886
APTT, s	30.6 (27.2–31.3)	28 (26.3–34.5)		0.815
INR	0.92 (0.9–1)	0.9 (0.9–1)		0.76
Creatinine, mg/dL	0.7 (0.6–0.8)	0.9 (0.8–1)		0.053
CK, U/L	152.5 (128.5–183.3)	122.5 (66.5–166.3)		0.187
**Glucose, mg/dL**	93.9 (89–95.3)	102 (98.5–106)		**0.009**
HbA1c, %	5.6 (5.2–5.7)	6 (5.6–6.1)		0.053
TSH, μIU/mL	1.8 (1.4–2.9)	1.5 (0.8–2.9)		0.656
NT-proBNP, pg/mL	95.4 (35.2–154.8)	81 (39.48–183)		0.721
Fibrinogen, µg/mL	48.5 (29.1–59)	51.4 (33.2–72.7)		0.909
FVIII, ng/mL	33.3 ± 22	37 ± 16.9	Welch’s *t*-test	0.666
PAI-I, pg/mL	848.8 (793.6–887.8)	882.3 (829.2–924.5)	M-W	0.689
PF-4, ng/mL	269 (258–285.5)	286 (272–295.5)		0.272
sP-selectin, ng/mL	39.8 ± 13	47.1 ± 8.7	Welch’s *t*-test	0.15

Abbreviations: ALT—alanine aminotransferase; APTT—activated partial thromboplastin time; AST—aspartate aminotransferase; CK—creatine kinase; HbA1c—glycated hemoglobin; HDLC—high-density lipoprotein cholesterol; HGB—hemoglobin; FVIII—coagulation factor VIII; IL-18—interleukin 18; INR—international normalized ratio; LDLC—low-density lipoprotein cholesterol; MPV—mean platelet volume; M-W—Mann–Whitney U test; NT-proBNP—N-terminal pro B-type natriuretic peptide; PAI-I—plasminogen activator inhibitor-1; PF-4—platelet factor-4; PLT—platelet count; PTX3—pentraxin 3; RBC—red blood cell count; sP-selectin—soluble P-selectin; TC—total cholesterol; TG—triglycerides; TSH—thyroid-stimulating hormone; WBC—white blood cell count.

## Data Availability

The original contributions presented in this study are included in the article. Further inquiries can be directed to the corresponding author.

## Abbreviations

The following abbreviations are used in this manuscript:

apoB	Apolipoprotein B
ALP	Alkaline phosphatase
ALT	Alanine aminotransferase
APTT	Activated partial thromboplatin time
AST	Aspartate aminotransferase
CD36	Cluster of differentiation 36
CD40L	Cluster of differentiation 40 ligand
CD62P	P-selectin
CK	Creatine kinase
DLCN	Dutch Lipid Clinic Network
DM	Diabetes mellitus
EGF-A	Epidermal growth factor homology domain A of LDLDR
ESC	European Society of Cardiology
GalNAc	Triantennary N-acetylgalactosamine
GGT	Gamma-glutamyl transferase
GPIIb/IIIa	Glycoprotein IIb/IIIa
FVIII	Coagulation factor VIII
HbA1c	Glycated hemoglobin
HDL-C	High-density lipoprotein cholesterol
HDL-c	High-density lipoprotein cholesterol concentration
HeFH	Heterozygous familial hypercholesterolemia
HGB	Hemoglobin
IL-18	Interleukin-18
INR	International normalized ratio
IQR	Interquartile range
LDL-C	Low-density lipoprotein cholesterol
LDL-c	Low-density lipoprotein cholesterol concentration
LDLR	Receptor for LDLC
LRP-1	LDL-related protein-1
MPV	Mean platelet volume
M-W	Mann–Whitney test
NT-proBNP	N-terminal prohormone B-type natriuretic peptide
NYHA	New York Heart Association
oxLDL	Oxidized LDL
PAI-1	Plasminogen activator inhibitor-1
PCSK9	Proprotein convertase subtilisin/kexin type 9
PCSK9i	Proprotein convertase subtilisin/kexin type 9 inhibitor
PCSK9 mabs	Monoclonal antibodies against PCSK9
PF-4	Platelet factor-4
PLT	Platelet count
RBC	Red blood cell count
RCTs	Randomized controlled trials
ROS	Reactive oxygen species
sCD62P	Soluble P-selectin
SD	Standard deviation
siRNA	Small interfering RNA
SMC	Smooth muscle cells
TC	Total cholesterol
TF	Tissue factor
TG	Triglycerides
TSH	Thyroid-stimulating hormone
WBC	White blood cell count
